# Metagenomic and metatranscriptomic analysis of human prostate microbiota from patients with prostate cancer

**DOI:** 10.1186/s12864-019-5457-z

**Published:** 2019-02-18

**Authors:** Ye Feng, Varune Rohan Ramnarine, Robert Bell, Stanislav Volik, Elai Davicioni, Vanessa M. Hayes, Shancheng Ren, Colin C. Collins

**Affiliations:** 1 Sir Run Run Shaw Hospital, Zhejiang University School of Medicine, Hangzhou, China; 2 Institute of Translational Medicine, Zhejiang University School of Medicine, Hangzhou, China; 3 Vancouver Prostate Centre, Vancouver, Canada; 4 GenomeDx Biosciences, Vancouver, Canada; 5 Genomics and Epigenetics Division, Garvan Institute of Medical Research, Darlinghurst, NSW Australia; 6 School of Health Systems and Public Health, University of Pretoria, Pretoria, South Africa; 7 St Vincent’s Clinical School, University of New South Wales, Randwick, NSW Australia; 8 Department of Urology, Shanghai Changhai Hospital, Second Military Medical University, Shanghai, China

**Keywords:** Metagenome, Metatranscriptome, Microbial infection, Prostate cancer, Pseudouridylation

## Abstract

**Background:**

Prostate cancer (PCa) is the most common malignant neoplasm among men in many countries. Since most precancerous and cancerous tissues show signs of inflammation, chronic bacterial prostatitis has been hypothesized to be a possible etiology. However, establishing a causal relationship between microbial inflammation and PCa requires a comprehensive analysis of the prostate microbiome. The aim of this study was to characterize the microbiome in prostate tissue of PCa patients and investigate its association with tumour clinical characteristics as well as host expression profiles.

**Results:**

The metagenome and metatranscriptome of tumour and the adjacent benign tissues were assessed in 65 Chinese radical prostatectomy specimens. *Escherichia*, *Propionibacterium*, *Acinetobacter* and *Pseudomonas* were abundant in both metagenome and metatranscriptome, thus constituting the core of the prostate microbiome. The biodiversity of the microbiomes could not be differentiated between the matched tumour/benign specimens or between the tumour specimens of low and high Gleason Scores. The expression profile of ten *Pseudomonas* genes was strongly correlated with that of eight host small RNA genes; three of the RNA genes may negatively associate with metastasis. Few viruses could be identified from the prostate microbiomes.

**Conclusions:**

This is the first study of the human prostate microbiome employing an integrated metagenomics and metatranscriptomics approach. In this Chinese cohort, both metagenome and metatranscriptome analyses showed a non-sterile microenvironment in the prostate of PCa patients, but we did not find links between the microbiome and local progression of PCa. However, the correlated expression of *Pseudomonas* genes and human small RNA genes may provide tantalizing preliminary evidence that *Pseudomonas* infection may impede metastasis.

**Electronic supplementary material:**

The online version of this article (10.1186/s12864-019-5457-z) contains supplementary material, which is available to authorized users.

## Background

Prostate cancer (PCa) is the most common malignant neoplasm among men in Western industrialized countries and its incidence is rapidly increasing in China. Globally there are 800,000 diagnoses and 300,000 deaths annually [1]. The aetiologies of the disease remain largely unknown. Since most precancerous and cancerous tissues show signs of inflammation, chronic bacterial prostatitis has been hypothesized to be a possible etiology [2–4]. In vitro cellular experiments have demonstrated that inflammation induced by *Escherichia coli* and *Propionibacterium acnes* can alter normal prostate epithelial cell differentiation and through this process inflammation accelerates the initiation of PCa with a basal cell origin [5–8]. Evidence also comes from epidemiological studies as up to 87% PCa patients have microbial DNA in their prostates. In particular, *P. acnes,* JC polyomavirus (JCV) and BK polyomavirus (BKV) have been more commonly detected in PCa patients than in controls [9–11].

Most of the above findings were obtained by performing traditional nucleic acid amplification tests, such as quantitative real-time PCR and amplification of 16S rRNA in concert with plasmid cloning and Sanger sequencing. The advent of next generation sequencing has revolutionized the study of human microbiomes. The body sites that have been intensely studied include gut, skin, oral cavity, vagina, but prostate has been largely overlooked. To date, only two studies have applied massively parallel 16S rRNA sequencing for investigating the prostate microbiome [12, 13]. While these two studies confirmed that the prostate was a non-sterile environment and discovered numerous bacterial organisms that had not been reported in prostate tissue, their limited sample size (< 20 patients) made it difficult to establish a reproducible link between microbial pathogens and PCa. Meanwhile, the innate flaws of the 16 s rRNA-based technologies, such as the amplification bias that may distort the bacterial composition, an inability to quantify the actual microbial load, as well as an inability to capture viruses that lack 16S rRNA, also preclude an accurate and complete characterization of prostate microbiome.

To address these limitations, we applied a shotgun-based integrated metagenomic and metatranscriptomic analysis to a cohort of 65 Chinese PCa patients. Moreover, the availability of host transcriptome offers the possibility to explore the interaction between the host cancerous tissue and its microbiome.

## Methods

### Patient selection and specimen processing

The information of patients and specimens is summarized in Additional file 1 [14]. Briefly, 65 PCa patients were recruited in this study, with an average age of 68.4 ± 7.3 years old. Treatment-naive prostate tumour and matched benign tissues were collected from the radical prostatectomy series at Shanghai Changhai Hospital and Fudan University Shanghai Cancer Center. The intact prostate glands were delivered to the pathology laboratory under sterile conditions. H&E slides of frozen human tumour tissues and their matched benign tissues were examined by a pathologist and a urology pathologist to confirm histological diagnosis and Gleason Score. The frozen sections were then used for DNA/RNA isolation.

### DNA and RNA sequencing

DNA and RNA preparation and library construction has been described [14]. In detail, for DNA library construction, DNA was extracted by phenol-chloroform and purified by the ethanol precipitation method. Then 2 μg of genomic DNA from each sample was fragmented using a Covaris Ultrasonicator®(Covaris, USA) to mean sizes of ~ 500 bp. After fragmentation, the purified, randomly fragmented DNA was treated with a mix of T4 DNA polymerase, Klenow fragments, T4 polynucleotide kinase and dNTPs for repairing the ends by blunting and phosphorylation. The blunted DNA fragments were subsequently 3′-adenylated using the Klenow (3′-5’exo) and ligated by T4 DNA ligase (Rapid) (Enzymatics, USA) to PE Index Adapters. After each step, the DNA was purified using the QIAquick PCR Purification Kit (Qiagen, Germany).

For RNA library construction, RNA was extracted using TRIzol reagent and then treated with DNase I, RNase-free (Thermo Fisher Scientific, USA). Ribosomal RNA was removed from total RNA by using Ribo-Zero® rRNA Removal Kit (Epicentre, USA). Then RNA was fragmented on Covaris Ultrasonicator® to mean sizes of ~ 200 bp, and the first cDNA strand was synthesized from fragmented RNA using PrimeScript® RT reagent Kit (Takara Bio, Japan). After purification with the Ampure XP Beads to remove dNTPs, second-strand synthesis was performed by incubation with RNase H, DNA polymerase, and dNTPs that contain dUTP in place of dTTP. A single 3′ ‘A’ base was added using Klenow (3′-5′ exo-) (Enzymatics, USA) and dATP to the end-repaired cDNA. Upon ligation with the PE Index Adaptors, the products were gel-recovered and subsequently digested with Uracil-N-Glycosylase (UNG) for removing the second-strand cDNA. Samples were then amplified by 15 cycles of PCR with Platinum Pfx DNA polymerase (Invitrogen, Thermo Fisher Scientific, USA).

All the constructed libraries were finally sequenced on HiSeq 2000 platform, producing 2 × 90-bp paired-end reads.

### Filtering human DNA and mapping to microbial genomes

Raw reads that contained adaptor sequences, too many Ns (> 10%) and/or low quality base (> 50% bases with quality < 5) were removed. Clean reads were aligned to human reference genome hg19 using the Burrows-Wheeler Aligner (BWA-MEM v0.7.5, http://bio-bwa.sourceforge.net/). PCR or sequencing optical duplicates were marked by Picard (v1.54, http://broadinstitute.github.io/picard).

The aligned reads were removed, and the unmapped reads were then mapped by BWA to National Center for Biotechnology Information (NCBI) full set of microbial reference genomes (Bacteria: https://www.ncbi.nlm.nih.gov/genome/microbes//; virus: https://www.ncbi.nlm.nih.gov/genomes/GenomesGroup.cgi?taxid=10239&host=human).

RepeatMasker (v4.0, http://www.repeatmasker.org) was used to identify repeat and low complexity reads. Any reads with three or more masked nucleotides were discarded for the next step. In order to further avoid false identification of bacterial reads which is mostly manifested as a large number of mapped reads being restricted to a few short genomic regions, the coverage uniformity of the bacterial genomes was assessed as described previously [15].

### Microbiome analyses

The taxonomic abundance was measured by the number of reads assigned to each microbial genus. We hypothesize that each human cell in the specimens have equal amount of human DNA/RNA. Accordingly, the purpose of normalization in this study was to minimize the bias caused by differences in sequencing coverage across samples and to make the human DNA/RNA comparable between specimens. In detail, for the metagenome analysis, a scaling normalization was done by multiplying the read counts by a constant so that each specimen has 1 × 10^9^ reads that were mapped to the human genome hg19. For the metatranscriptome analysis, a scaling normalization was done by multiplying the read counts by a constant so that each specimen has 1 × 10^4^ reads that were mapped to the housekeeping gene GAPDH (ENSG00000111640). The numbers of raw and normalized reads are listed in Additional file 2.

The indices of alpha- and beta- diversity were calculated using the QIIME package (v1.9, http://qiime.org/). Comparison of these indices between groups was conducted using Student’s t-test. The Non-Metric Multi-Dimensional Scaling (NMDS) analysis was performed using the vegan package in R software v3.3.

Paired comparisons of the read counts between tumour and benign specimens were conducted using Wilcoxon matched-pairs signed rank test. The *p*-values were corrected for false discovery using the Benjamini & Hochberg (BH) method. Only the genera with BH-adjusted *p*-value < 0.05 and fold-change > 2 were considered as significantly different between specimen groups.

### Correlation analysis of bacterial and host expression

Nonparametric Spearman correlation was calculated between the bacterial genes, genera and the host genes as well as the clinical parameters, including Gleason Score and prostate-specific antigen (PSA) level, in terms of their values among the 130 specimens. The returned *p*-values were corrected for false discovery using BH method. A Spearman correlation coefficient > 0.7 as well as a BH-adjusted *p*-value < 0.05 was taken as the threshold for a significantly strong relationship.

The sequences of the bacterial genes were obtained by de novo assembly using Trinity software (v2.4, https://github.com/trinityrnaseq/trinityrnaseq/wiki). Annotation of these transcripts was performed by BLAST search of the assembled nucleotide sequences against NCBI non-redundant protein library. The read counts of these bacterial genes were normalized with the same method as the metatranscriptome analysis.

### Kaplan-Meier (KM) survival analysis

The Cleveland Clinic Foundation (CCF) cohort [16] and the JHMI cohort [17] were used for the KM analysis. The patients in the cohorts were split into two subgroups by the mean expression value of the three small RNA genes, respectively, and then the Kaplan-Meier survival analysis was performed using R package “survival” (http://cran.r-project.org/web/packages/survival/index.html). Weighted Cox regression models (survival 2.38–3) were used to generate KM curve *p*-values.

## Results

### Microorganisms identified in prostate

The cohort in this study is comprised of 65 matched PCa tumours and adjacent benign tissue from Chinese patients who underwent radical prostatectomy. Whole genome sequencing yielded 176–330 Gb between specimens, and whole transcriptome sequencing ranged from 5 to 22 Gb. After filtering human RNA and mapping against microbial genomes, the abundance of bacterial reads derived from metagenome varied from 69 to 101,542 reads per 10^9^ human reads; accordingly, the multiplicity of infection (MOI) varied from 6.8 × 10^− 5^ to 0.1 bacterial cells per human cell when assuming an average size of bacterial genomes to be 3 Mb. Similarly, most reads derived from metatranscriptome were also of human origin (> 97%), with the bacterial abundance ranging from 3.9 × 10^3^ to 3.0 × 10^6^ reads per 10^4^ host GAPDH reads.

**Fig. 1:**
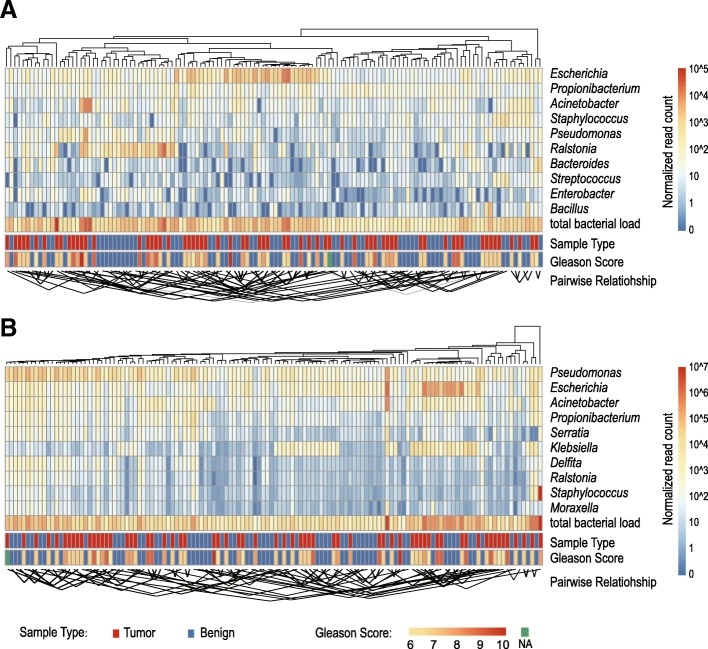
Bacterial composition of prostate microbiome revealed by metagenomic (**a**) and metatranscripotomic sequencing (**b**). The upper UPGMA tree was constructed based on the weighted_UniFrac distance between specimens. The heatmap below represents the normalized read counts for the top 10 abundant genera. The lines below connect the matched tumour/benign specimens

A total of 47 and 116 bacterial genera were identified in the metagenome and metatranscriptome, respectively, with 43 genera being present in both (Additional file 3). The most abundant genera identified from both metagenome and metatranscriptome included *Escherichia*, *Propionibacterium*, *Acinetobacter* and *Pseudomonas* (Fig. 1), although the exact quantitative compositions varied (Fig. 1 and Additional file 3).

Four viruses were identified in the metagenome and metatranscriptome, all of which belong to dsDNA viruses. However, for majority of specimens, the viral read counts were close to zero and therefore uninformative (Additional file 4).

### Comparison of biodiversity indices between specimen groups

**Fig. 2:**
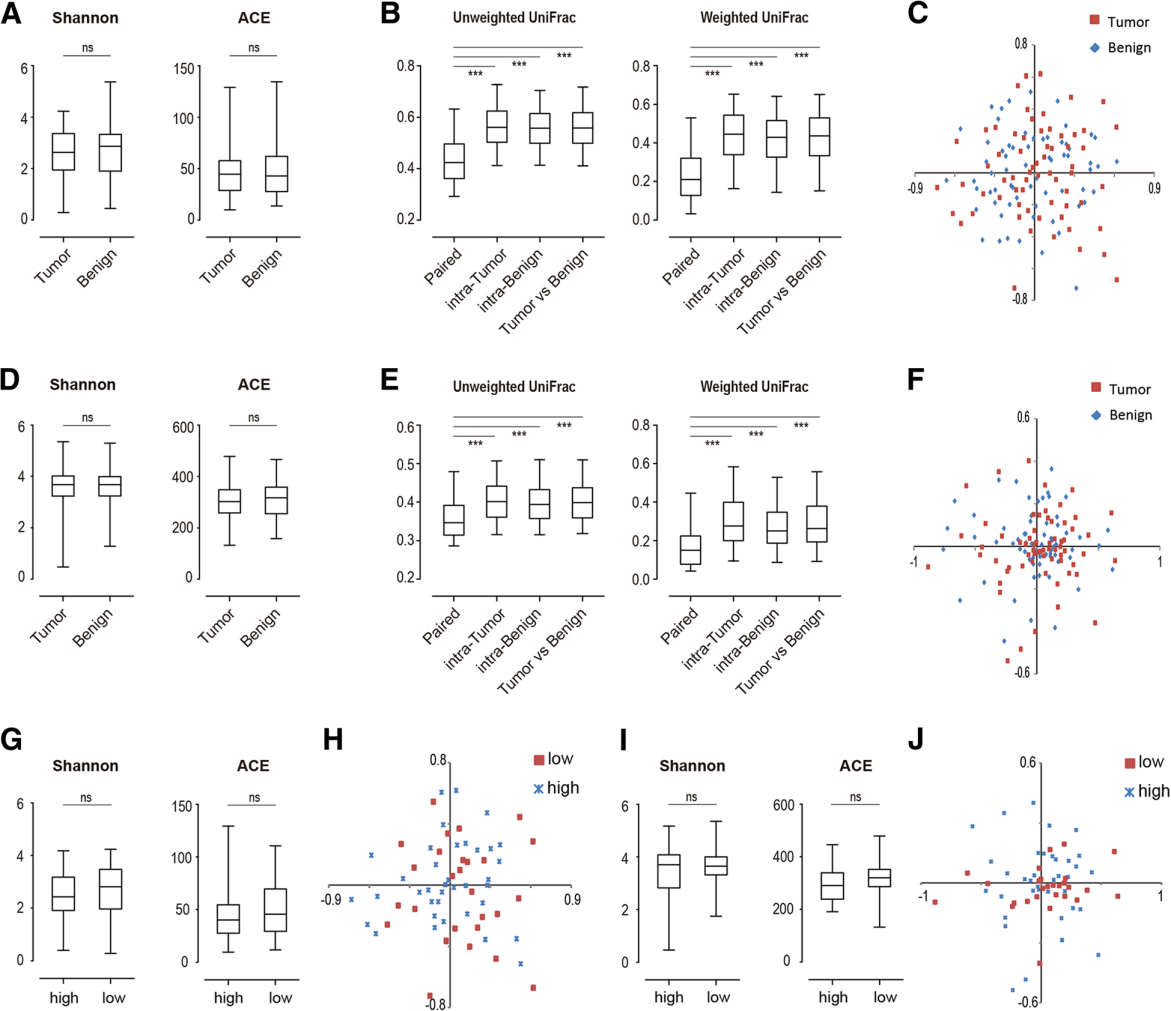
Comparison of biodiversity indices between different groups of specimens. Panel **a**-**f** show comparisons between tumour and benign specimens; panel **g**-**j** show comparisons between specimens of high and low Gleason Scores. Panel **a**-**c** and **g**-**h** are derived from metagenomic data; panel **d**-**f** and **i**-**j** are derived from metatranscriptomic data. Panel **a**, **d**, **g** and **i** show comparison of alpha-diversity indices; ns (not significant), *p* > 0.05 by unpaired Student’s t-test. Panel **b** and **e** show comparisons of beta-diversity indices. Distances between specimens were divided into four groups: ‘paired’, distance between the matched tumour/benign specimens from the same patients; ‘intra-tumour’, distance between tumour specimens from different patients; ‘intra-benign’, distance between benign specimens from different patients; ‘Tumour vs Benign’, distance between tumour and benign specimens from different patients. ***, *p* < 0.001 by unpaired Student’s t-test. Panel **c**, **f**, **h** and **j** are NMDS plots

At both metagenomic and metatranscriptomic level, the tumour and benign specimens did not differ from each other in terms of the alpha-diversity of their microbiomes (paired t test, *p* > 0.05; Fig. 2a, d). The beta-diversity showed that the paired specimens from the same patients had a more similar bacterial composition to each other than between different patients (paired t test, *p* < 0.05; Fig. 2b, e); the NMDS analysis could not separate tumour and benign specimens (Fig. 2c, f).

Neither the total bacterial load nor any specific genus showed significant differential distribution between the tumour and benign specimens at the metagenomic level (Wilcoxon signed-rank test, *p* > 0.05). The situation at the metatranscriptomic level was the same.

We further divided the patients into lower and higher grade groups based on Gleason Score: the low-grade group included 6 and 3 + 4; the higher-grade group included 8, 9, 10 and 4 + 3. The alpha-diversity did not differ significantly between these two groups (paired t test, *p* > 0.05; Fig. 2g, i), and the NMDS analysis could not separate them (Fig. 2h, j).

### Correlation of microbiome with host expression profile

**Fig. 3:**
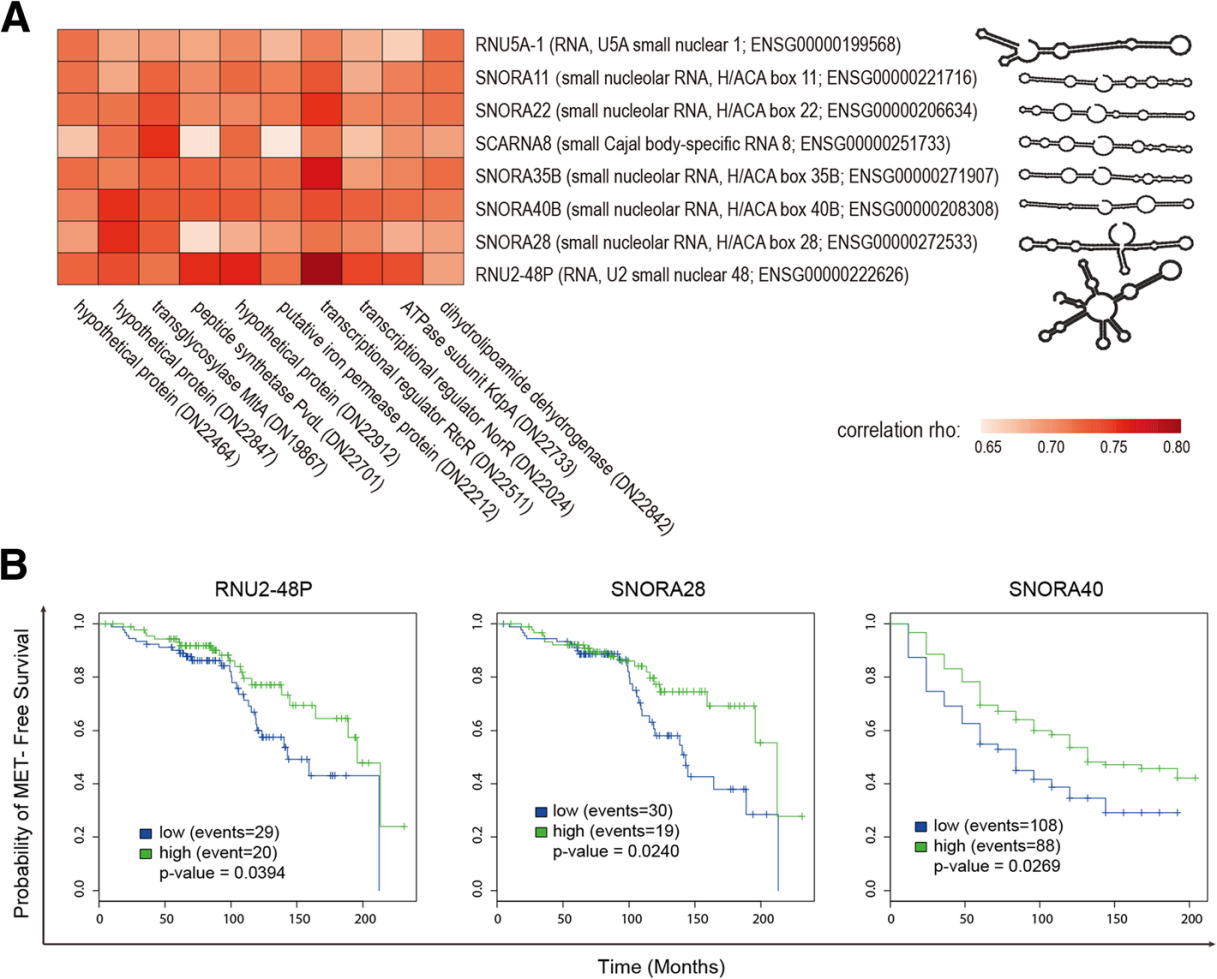
Correlation between bacterial metatranscriptome and host gene expression. Panel **a** shows the Spearman correlation values between ten *Pseudomonas* genes (listed below) and eight host genes (listed right). The predicted secondary structures for these host genes are displayed. The detailed annotation and nucleotide sequences of these bacterial genes are listed in Additional file 5. The Kaplan-Meier (KM) plots in panel **b** categorize the patients into the low and high group based on a median split of expression of the three small RNA genes. The higher the probability on the y-axis, the higher the chance of these patients NOT having metastatic recurrence (MET). The *p*-value of KM curve was generated by Weighted Cox regression models. The analysis for RNU2-48P and SNORA28 used the Cleveland Clinic Foundation (CCF) cohort [16], and the analysis for SNORA40 used the JHMI cohort [17]

At either metagenomic or metatranscriptomic level, no significant correlation could be found between the bacterial load (either total or of any specific genus) and any clinical parameters such as Gleason Score and prostate-specific antigen (PSA) level (Spearman correlation test, r < 0.7). Correlations between microbial and host genes in terms of their expression profile were also investigated. Interestingly, we identified 191 pairs of host-pathogen genes that have a significantly similar expression profile (Spearman correlation test, r > 0.7), nearly half of which involved ten *Pseudomonas* genes and eight host genes (Fig. 3). All eight host genes encode small RNA and share remarkably similar structures. Moreover, differential expression of SNORA28, RNU2-48P and SNORA40B stratified patients in terms of metastatic recurrence in another two well-studied cohorts [16, 17], suggesting that the infections caused by *Pseudomonas spp.* and the associated expression of these small RNAs are negatively related with metastasis.

## Discussion

This is the first study employing an integrated metagenomic and metatranscriptomic approach to investigate human prostate microbiome. *Escherichia*, *Propionibacterium*, *Acinetobacter*, and *Pseudomonas* were found to be abundant in both the metagenome and metatranscriptome and thus constitute the core of the prostate microbiome in this Chinese population. *Escherichia* and *Propionibacterium* have previously been shown to stimulate progression of PCa in in vitro experiments [6, 8, 9], but the biological significance for the other bacteria identified in this study remains unclear. As expected, many of the organisms identified in the prostate tissue have also been detected in urine, semen, and expressed prostatic secretions [18–21]. This similar microbial composition provides evidence for the theory of ascending transmitted infection in the male urogenital tract to the seminal vesicles and prostate.

Several previous studies have associated a history of sexually transmitted infections with the risk of prostate cancer [22–24]. Nevertheless, we did not detect sexually transmitted disease-related organisms in this study, such as *Neisseria gonorrhoeae*, *Chlamydia trachomatis*, *Mycoplasma hominis* or *Ureaplasma urealyticum*. Further, we did not detect viruses reported to be present in prostate tissues such as JCV, BKV, human papillomavirus (HPV) or human cytomegalovirus (HCMV) [11, 25–27]. Thus untreated sexually transmitted infections or viral infections are not expected to account for a significant proportion of PCa risk in the general male population, or at least in the investigated cohort.

The most urgent question in this field is whether the microbiomes from the tumour and benign tissues differ from each other and whether this difference has a causal relationship with carcinogenesis. In this study, we did not find any bacterial species that showed significantly differential distribution between tumours and their matched benign specimens at either DNA or RNA level. Also we did not find a significant difference between the tumour specimens of low and high Gleason Scores. These findings suggest that the identified microbiota may comprise the normal flora of the prostate. Due to the close proximity of the regions compared and the field effect, we still cannot entirely exclude a causal role for bacterial infection and local PCa progression. To fully address this question, non-diseased prostate specimens are required to determine whether a healthy prostate is normally sterile or has a specific microbial flora.

The availability of the host transcriptome allowed us to detect a correlation between expression of human small RNA genes and *Pseudomonas* genes. Functional annotation has assigned these small RNAs to pseudouridylation, which is a major form of post-transcriptional RNA modification. Through pseudouridylation these small RNAs participate in the regulation of gene expression and therefore affect pre-mRNA splicing, translation fidelity and possibly mRNA stability and decay [28, 29]. It has been established that pseudouridylation can be induced as a response to external stress, such as heat shock or nutrient deprivation [30]. High expression of these small RNAs in a subset of patients with low rates of metastasis suggests a negative association between *Pseudomonas* infection and metastasis. If this association is validated in larger cohorts then the expression profile of the bacterial and small RNA genes may be used as biomarker for active surveillance.

## Conclusion

In this study we employed an integrated metagenomic and metatranscriptomic approach to investigate the prostate microbiome of PCa patients. Bacteria were detected in all specimens but the composition was not significantly different between the matched tumour and adjacent benign tissues or between different tumour grades. We also identified a strongly correlated expression profile between *Pseudomonas* genes and human small RNAs that may be related to metastasis. The exact mechanism of this host-pathogen interaction awaits future research.

## Additional files

Additional file 1: Clinical pathological information of study cohort. (XLSX 14 kb)

Additional file 2: The number of raw and normalized reads for each taxonomic unit for both metagenome and metatranscriptome. (XLSX 337 kb)

Additional file 3: Comparison of bacterial composition between metagenome and metatranscriptome. (A) Venn diagram of bacterial genera identified by metagenome, metatranscriptome and the study by Yow et al. [13]. (B) The NMDS plot shows that the metagenomic and metatranscriptomic data could be clearly separated in terms of bacterial composition. Each dot represented a specimen. (TIF 217 kb)

Additional file 4: Prostatic virome. The heatmap represents the normalized read counts for the identified viruses. (TIF 236 kb)

Additional file 5: Detailed annotation and nucleotide sequences of the 10 bacterial genes that have the correlated expression profile with the eight small RNA genes. (XLSX 25 kb)

## Abbreviations

BH: Benjamini & Hochberg
BKV: BK polyomavirus
BWA: Burrows-Wheeler Aligner
CCF: Cleveland Clinic Foundation
HCMV: Human cytomegalovirus
HPV: Human papillomavirus
JCV: JC polyomavirus
KM: Kaplan-Meier
MET: Metastatic recurrence
MOI: Multiplicity of infection
NMDS: Non-Metric Multi-Dimensional Scaling
PCa: Prostate cancer
