# Supplementary figures and images for: Metagenomic and metatranscriptomic analysis of human prostate microbiota from patients with prostate cancer
Source: BMC Genomics. 2019 Feb 18;20:146. doi: 10.1186/s12864-019-5457-z (PMC6379980; doi:10.1186/s12864-019-5457-z)

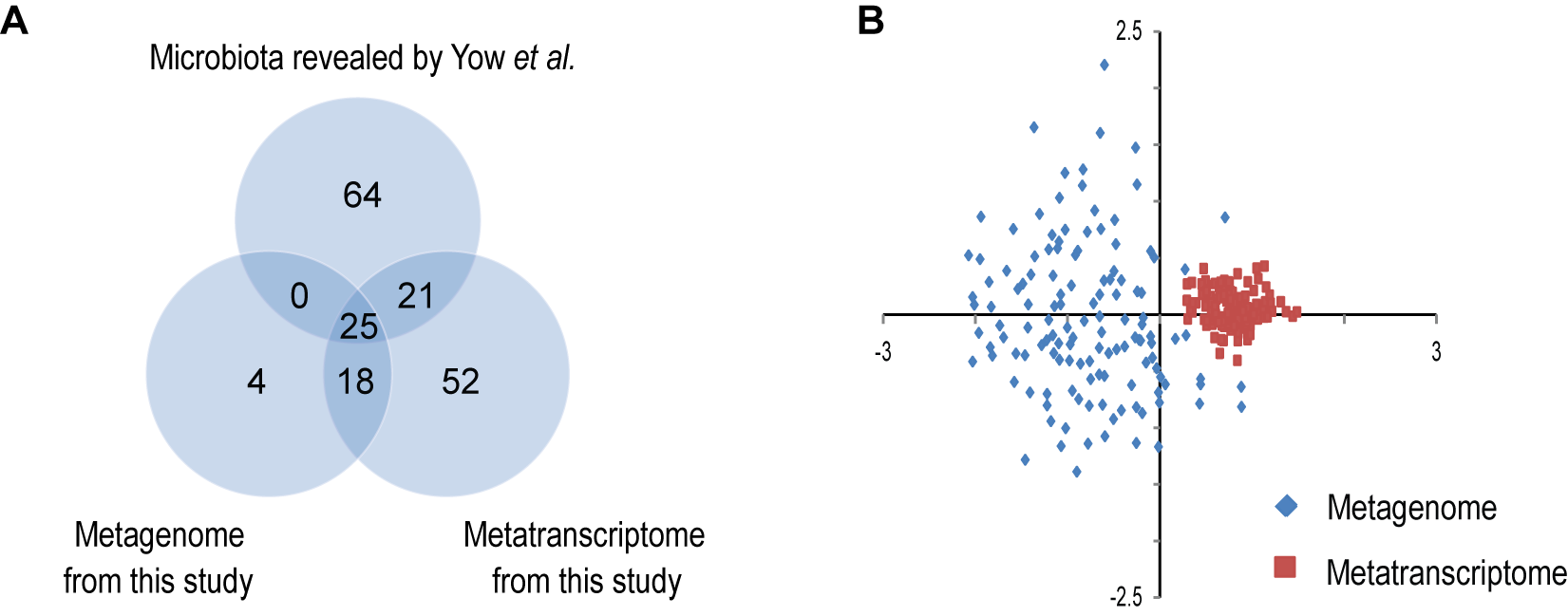

Supplement: Supplementary file 3 — Comparison of bacterial composition between metagenome and metatranscriptome. (A) Venn diagram of bacterial genera identified by metagenome, metatranscriptome and the study by Yow et al. [13]. (B) The NMDS plot shows that the metagenomic and metatranscriptomic data could be clearly separated in terms of bacterial composition. Each dot represented a specimen. (TIF 217 kb) [file 12864_2019_5457_MOESM3_ESM.tif]

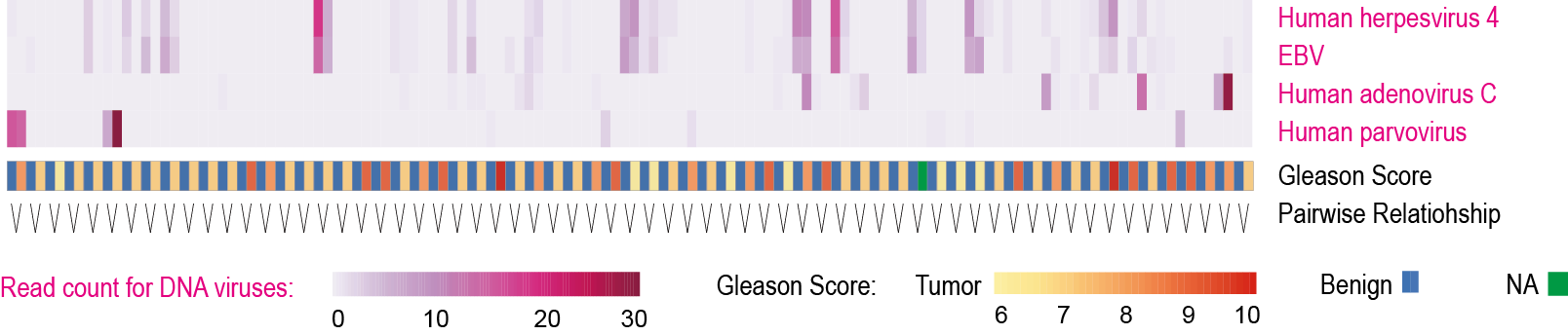

Supplement: Supplementary file 4 — Prostatic virome. The heatmap represents the normalized read counts for the identified viruses. (TIF 236 kb) [file 12864_2019_5457_MOESM4_ESM.tif]
